# Supplementary material for: Pyruvate Dehydrogenase Kinase 1 inhibition mediated oxidative phosphorylation enhancement in cartilage promotes osteoarthritis progression
Source: BMC Musculoskelet Disord. 2023 Jul 20;24:597. doi: 10.1186/s12891-023-06585-6 (PMC10357736; doi:10.1186/s12891-023-06585-6)
Supplement: Supplementary file 1 — Additional file 1. [file 12891_2023_6585_MOESM1_ESM.zip › BP.Gsea.1653623667859/GOBP_MITOTIC_CYTOKINETIC_PROCESS.html]

Details for gene set GOBP\_MITOTIC\_CYTOKINETIC\_PROCESS[GSEA]

|  || Dataset | 55457\_collapsed\_to\_symbols.cls.cls #OA\_versus\_normal.cls.cls #OA\_versus\_normal\_repos |
| Phenotype | cls.cls#OA\_versus\_normal\_repos |
| Upregulated in class | OA |
| GeneSet | GOBP\_MITOTIC\_CYTOKINETIC\_PROCESS |
| Enrichment Score (ES) | 0.4669988 |
| Normalized Enrichment Score (NES) | 1.4804978 |
| Nominal p-value | 0.054466233 |
| FDR q-value | 0.37207678 |
| FWER p-Value | 1.0 |
Table: GSEA Results Summary

  

Fig 1: Enrichment plot: GOBP\_MITOTIC\_CYTOKINETIC\_PROCESS      
 Profile of the Running ES Score & Positions of GeneSet Members on the Rank Ordered List

  

| SYMBOL | TITLE | RANK IN GENE LIST | RANK METRIC SCORE | RUNNING ES | CORE ENRICHMENT || 1 | CHMP2B | charged multivesicular body protein 2B [Source:HGNC Symbol;Acc:HGNC:24537] | 129 | 0.876 | 0.1474 | Yes |
| 2 | CHMP4A | charged multivesicular body protein 4A [Source:HGNC Symbol;Acc:HGNC:20274] | 526 | 0.649 | 0.2332 | Yes |
| 3 | CHMP5 | charged multivesicular body protein 5 [Source:HGNC Symbol;Acc:HGNC:26942] | 556 | 0.638 | 0.3456 | Yes |
| 4 | CHMP2A | charged multivesicular body protein 2A [Source:HGNC Symbol;Acc:HGNC:30216] | 1269 | 0.464 | 0.3737 | Yes |
| 5 | CHMP6 | charged multivesicular body protein 6 [Source:HGNC Symbol;Acc:HGNC:25675] | 2140 | 0.345 | 0.3682 | Yes |
| 6 | KIF20B | kinesin family member 20B [Source:HGNC Symbol;Acc:HGNC:7212] | 2714 | 0.278 | 0.3738 | Yes |
| 7 | CHMP3 | charged multivesicular body protein 3 [Source:HGNC Symbol;Acc:HGNC:29865] | 2733 | 0.277 | 0.4221 | Yes |
| 8 | VPS4B | vacuolar protein sorting 4 homolog B [Source:HGNC Symbol;Acc:HGNC:10895] | 2783 | 0.271 | 0.4670 | Yes |
| 9 | PDCD6IP | programmed cell death 6 interacting protein [Source:HGNC Symbol;Acc:HGNC:8766] | 4956 | 0.076 | 0.3120 | No |
| 10 | CEP55 | centrosomal protein 55 [Source:HGNC Symbol;Acc:HGNC:1161] | 5787 | 0.009 | 0.2492 | No |
| 11 | KIF20A | kinesin family member 20A [Source:HGNC Symbol;Acc:HGNC:9787] | 6457 | -0.039 | 0.2043 | No |
| 12 | NUP62 | nucleoporin 62 [Source:HGNC Symbol;Acc:HGNC:8066] | 6800 | -0.063 | 0.1891 | No |
| 13 | ARF1 | ADP ribosylation factor 1 [Source:HGNC Symbol;Acc:HGNC:652] | 7152 | -0.092 | 0.1784 | No |
| 14 | CHMP1B | charged multivesicular body protein 1B [Source:HGNC Symbol;Acc:HGNC:24287] | 7339 | -0.105 | 0.1828 | No |
| 15 | RHOA | ras homolog family member A [Source:HGNC Symbol;Acc:HGNC:667] | 8601 | -0.201 | 0.1211 | No |
| 16 | CHMP7 | charged multivesicular body protein 7 [Source:HGNC Symbol;Acc:HGNC:28439] | 9723 | -0.294 | 0.0869 | No |
| 17 | VPS4A | vacuolar protein sorting 4 homolog A [Source:HGNC Symbol;Acc:HGNC:13488] | 10768 | -0.401 | 0.0779 | No |
| 18 | CHMP1A | charged multivesicular body protein 1A [Source:HGNC Symbol;Acc:HGNC:8740] | 11401 | -0.487 | 0.1164 | No |
Table: GSEA details [plain text format]

  

Fig 2: GOBP\_MITOTIC\_CYTOKINETIC\_PROCESS      
 Blue-Pink O' Gram in the Space of the Analyzed GeneSet

  

Fig 3: GOBP\_MITOTIC\_CYTOKINETIC\_PROCESS: Random ES distribution      
 Gene set null distribution of ES for **GOBP\_MITOTIC\_CYTOKINETIC\_PROCESS**
